# Resetting the immune setpoint of cold tumors through a ferroptosis–cGAS–STING amplification circuit

**DOI:** 10.1186/s12967-026-08683-0

**Published:** 2026-07-23

**Authors:** Yi Wang, Xinru Li, Yuan Liang, Yawen Li, Yixin Zhao, Pengna Guo, Yuguang Zhao

**Affiliations:** Cancer Center, The First Hospital of Jilin University, No. 71 Xinmin Street, Chaoyang District Changchun, Changchun, Jilin 130021 China

**Keywords:** Ferroptosis, cGAS-STING pathway, Tumor immune microenvironment, Cold tumors, Immunotherapy

## Abstract

Cold tumors remain a major challenge in cancer immunotherapy because of insufficient immune cell infiltration and limited inflammatory signaling, resulting in poor responses to immune checkpoint inhibitors. However, accumulating evidence suggests that many cold tumors are not completely immunologically inert but instead persist in a low-level immune state that fails to sustain effective immune amplification. Based on this perspective, we propose that cold tumors can be more appropriately understood as a threshold-constrained immune state, in which antitumor immune responses have been initiated but remain unable to surpass the immune activation threshold required to establish sustained immune amplification. Within this conceptual framework, we further propose a hierarchical model of immune dysfunction consisting of priming failure, trafficking failure, and penetration failure to explain the systemic mechanisms underlying immune resistance in cold tumors. Building upon this framework, we introduce the Ferroptosis–cGAS–STING amplification circuit as a conceptual model for driving immune state transition. In this model, ferroptosis activates the cGAS–STING–type I interferon axis through the release of tumor antigens and DNA-derived danger signals, whereas STING signaling, in turn, enhances cellular susceptibility to ferroptosis, thereby forming a transient amplification module constrained by temporal and metabolic conditions. This amplification module may enable subthreshold immune responses to overcome the immune activation threshold, thereby creating conditions for subsequent immune amplification while coordinately improving immune priming, immune cell recruitment, and tumor penetration, ultimately alleviating hierarchical immune barriers. Furthermore, we define the functional reprogramming of the tumor–immune system driven by crossing the immune activation threshold as immune setpoint resetting. We also discuss the key regulatory determinants and biological boundaries of this conceptual framework, including the temporal window of amplification, signal intensity, spatial constraints, and functional heterogeneity across different tumor contexts, together with its current limitations and future research directions. Overall, this review establishes a unified conceptual framework linking ferroptosis, innate immune activation, and immune remodeling in cold tumors, providing a systems-level perspective for understanding immune state transition and a theoretical foundation for designing immune interventions based on threshold crossing.

## Introduction

In recent years, immune checkpoint inhibitors (ICIs) have fundamentally reshaped the therapeutic landscape of multiple malignancies by blocking inhibitory pathways such as programmed cell death protein 1/programmed death-ligand 1 (PD-1/PD-L1) and restoring CD8⁺ T cell effector function [1]. In tumors such as melanoma, non-small cell lung cancer, and clear cell renal cell carcinoma, ICIs have achieved durable clinical benefits [2–4]. However, a substantial proportion of patients still exhibit limited responses to immunotherapy, indicating that the release of immune inhibition alone does not necessarily translate into effective and sustained antitumor immunity. How to establish durable immune amplification in tumors with insufficient immune responses therefore remains a central challenge in cancer immunotherapy.

This challenge is particularly evident in so-called cold tumors, which are typically characterized by limited immune cell infiltration and weak inflammatory signaling. Traditionally, cold tumors have been classified according to the spatial distribution of CD8⁺ T cells, including the immune-desert and immune-excluded phenotypes [5]. Although these classifications capture histological characteristics, they remain largely descriptive and fail to explain why immune activity in these tumors cannot progress into a sustained and self-amplifying cancer–immunity cycle [6]. Increasing evidence suggests that cold tumors are not completely immunologically silent; rather, they retain a certain degree of antigenicity and sporadic immune activity, yet these signals remain insufficient to initiate a durable and self-reinforcing antitumor immune response.

Based on this perspective, we propose that cold tumors are more appropriately understood as a threshold-constrained immune state. Within this framework, the failure of antitumor immunity is not caused by the absence of immune signals, but rather by the inability of these signals to surpass the immune activation threshold required to establish effective immune amplification. Furthermore, this immune dysfunction is unlikely to result from a single defect but instead reflects the cumulative failure of multiple sequential processes. According to their functional roles in antitumor immunity, these constraints can be conceptualized as three hierarchical layers: priming failure, trafficking failure, and penetration failure. Together, these layered defects restrict the progressive amplification of immune responses and collectively elevate the immune activation threshold required for effective antitumor immunity.

Within a threshold-constrained immune state, mechanisms capable of driving immune responses across the immune activation threshold generally require three fundamental features: (1) effective antigen exposure together with danger-signal input; (2) coupling of cytosolic DNA sensing to a systemic immune amplification program; and (3) a regulatable positive-feedback or metabolic coupling mechanism capable of sustaining and stabilizing immune responses.

From the perspective of immune initiation, different forms of regulated cell death differ substantially in their capacity to activate antitumor immunity. Apoptosis is generally considered immunologically silent because cellular integrity is largely preserved during non-inflammatory clearance, thereby providing limited antigen exposure and danger-signal input [7]. Although necroptosis and pyroptosis can induce local inflammatory responses, their immunological effects are primarily restricted to inflammatory cascade amplification and generally lack the capacity to initiate sustained, cytosolic DNA sensing-driven systemic immune amplification [8]. Consequently, neither fully satisfies the above requirements. In contrast, ferroptosis is frequently accompanied by the release of mitochondrial DNA (mtDNA) and the exposure of damage-associated molecular patterns (DAMPs), thereby simultaneously providing antigenic signals and DNA-derived danger signals that serve as a critical input for immune priming [9].

In the context of cytosolic DNA sensing and immune amplification, not all signaling pathways are equally capable of driving durable antitumor immune remodeling. AIM2 primarily mediates IL-1β/IL-18 release and pyroptosis through inflammasome activation, exerting predominantly local inflammatory effects with limited capacity to induce type I interferon production or drive systemic T cell immune remodeling [10]. Although TLR9 can induce type I interferon responses, its expression is restricted to specific immune cell subsets and its endosomal localization indicates that it is not a primary pathway for sensing endogenous cytosolic DNA in tumor cells [11]. By contrast, the cyclic GMP–AMP synthase (cGAS)–stimulator of interferon genes (STING) pathway serves as a central hub linking cytosolic DNA sensing to systemic immune amplification. Activation of this pathway induces type I interferon (IFN-I) signaling, promotes dendritic cell (DC) maturation, and enhances chemokine-mediated recruitment of CD8^+^T cells, thereby transforming tumor-derived danger signals into systemic antitumor immune remodeling [12].

Based on these mechanistic characteristics, among the potential combinations of regulated cell death pathways and DNA-sensing pathways, the Ferroptosis–cGAS–STING axis most comprehensively fulfills the sequential requirements of DNA-derived signal input, IFN-I-mediated immune amplification, and immune–metabolic feedback. Moreover, this axis is currently supported by the most coherent mechanistic evidence and the most consistent preclinical findings, making it the primary focus of this Review. Although functional coupling between other forms of regulated cell death and DNA-sensing pathways may also contribute to immune regulation under specific tumor contexts, these interactions are generally more context-dependent and currently lack sufficient evidence to support an equally integrated threshold-crossing amplification framework.

Accordingly, we focus on the potential functional coupling between ferroptosis and the cGAS–STING pathway and propose the concept of the Ferroptosis–cGAS–STING amplification circuit. In this model, ferroptosis provides antigenic and DNA-derived inputs that activate innate immune sensing, whereas STING-dependent IFN-I signaling reciprocally enhances oxidative vulnerability and creates conditions favorable for further immune amplification. We propose that the transient coupling between ferroptotic stress and innate immune signaling enables immune responses that would otherwise remain below the immune activation threshold to cross this critical boundary, thereby providing a potential mechanistic basis for immune setpoint resetting within the tumor–immune system [13].

## Cold tumors as a threshold-constrained immune state

### Understanding cold tumors as a threshold-constrained immune state

Traditionally, cold tumors have been defined according to the spatial distribution of CD8⁺ T cells within tumor tissues, including the immune-desert and immune-excluded phenotypes [5]. Although these classifications are useful for describing histopathological characteristics, they remain largely phenotypic and provide limited insight into why immune responses in these tumors fail to become effectively activated and sustained.

Based on this limitation, we propose a functional definition in which cold tumors are conceptualized as a threshold-constrained immune state. In this state, antitumor immune responses are not completely absent but instead persist at a low intensity that is insufficient to sustain itself. Consequently, the immune system remains outside the immune amplification program and fails to cross the critical boundary required to engage a self-reinforcing positive-feedback immune response.

From a systems dynamics perspective, this critical boundary can be understood as the transition point at which immune responses shift from localized signaling to systemic immune amplification. Functionally, it reflects the integrated output of multiple key processes within the cancer–immunity cycle. In this Review, we operationally define this boundary as the immune activation threshold, namely the minimum integrated level of immune activation required to initiate and sustain a self-reinforcing antitumor immune response. Rather than being determined by a single molecular event, this threshold is collectively governed by multiple key parameters, including antigen presentation efficiency, IFN-I signaling intensity, and effector CD8⁺ T cell infiltration. Together, these factors determine whether the immune system possesses the capacity to enter a sustained positive-feedback amplification program [6]. When one or more of these processes remain insufficient, the overall immune response is maintained in a low-level steady state and therefore fails to achieve threshold crossing.

### Hierarchical immune failure in cold tumors: priming, trafficking, and penetration

Within a threshold-constrained immune state, immune failure does not arise from a single defect but rather reflects the combined effects of multiple stage-specific barriers [6]. Based on the functional organization of the antitumor immune response, these constraints can be integrated into a hierarchical immune failure model comprising three interrelated layers: priming failure, trafficking failure, and penetration failure.

Priming failure refers to impairment of adaptive immune initiation. In many cold tumors, although tumor antigens are present, insufficient antigen release, inadequate dendritic cell (DC) maturation signals, or inefficient cross-presentation limit the expansion of tumor-specific CD8⁺ T cells [14, 15]. As the first step of the antitumor immune response, impaired priming often constitutes a critical bottleneck that restricts subsequent immune amplification.

Trafficking failure occurs during immune cell migration. Even when tumor-specific CD8⁺ T cells are successfully activated in peripheral lymphoid organs, their recruitment to the tumor site depends on effective chemokine gradients. The CXCL9/CXCL10–CXCR3 axis plays a central role in directing effector CD8⁺ T cell homing [16, 17]. When this signaling axis is insufficient, activated CD8⁺ T cells fail to efficiently infiltrate the tumor microenvironment, thereby preventing the establishment of effective local immune pressure.

Penetration failure reflects spatial barriers within the tumor microenvironment. In certain immune-excluded tumors, effector CD8⁺ T cells can reach the tumor margin but are unable to penetrate the tumor parenchyma. This phenomenon is commonly associated with cancer-associated fibroblast (CAF)-mediated extracellular matrix (ECM) deposition, transforming growth factor-β (TGF-β)-driven stromal remodeling, and aberrant vascular architecture, which collectively establish physical and functional barriers that restrict immune cell infiltration [18–20].

**Fig. 1:**
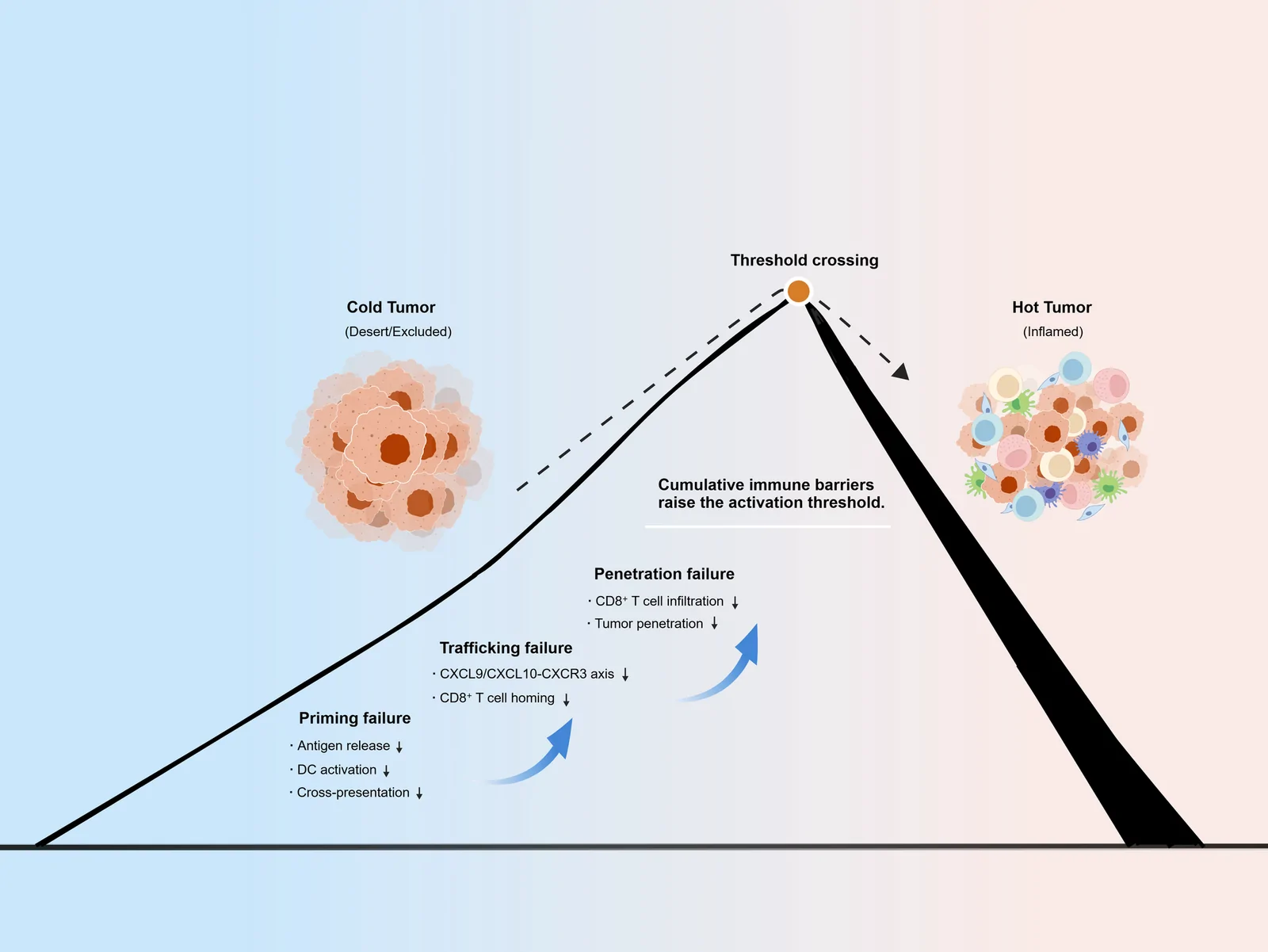
Cold tumors as a threshold-constrained immune state with hierarchical barriers. This figure illustrates the concept that cold tumors are restricted by an “immune activation threshold” during the transition toward immune-active (hot) tumors. Rather than being completely immune silent, cold tumors exist in a low-intensity immune state that fails to sustain self-amplifying responses because immune activity does not cross the activation threshold. Antitumor immunity is constrained by the cumulative effects of multilayered barriers, including: (1) priming failure, characterized by insufficient antigen release, limited dendritic cell (DC) activation, and impaired cross-presentation; (2) trafficking failure, characterized by inadequate CXCL9/CXCL10–CXCR3 signaling and defective CD8⁺ T cell recruitment; and (3) penetration failure, characterized by restricted CD8⁺ T cell infiltration into the tumor parenchyma. These hierarchical constraints collectively elevate the immune activation threshold, maintaining the tumor in a subthreshold immune state. Only when immune signals exceed this threshold can the tumor microenvironment transition into an inflamed “hot” state capable of sustaining immune amplification

Importantly, these three layers of immune failure are not independent but instead exhibit hierarchical coupling. Defects in upstream priming constrain downstream CD8⁺ T cell recruitment, whereas insufficient chemokine signaling and structural barriers further amplify immunosuppressive effects. The cumulative impact of these multilevel constraints collectively elevates the immune activation threshold, thereby maintaining tumors in a low-immune steady state. Thus, this hierarchical model not only captures the structural organization of immune failure in cold tumors but also defines the functional constraints that maintain the threshold-constrained immune state (Fig. 1).

### Immune checkpoint blockade releases suppression but does not lower the activation threshold

ICIs restore antitumor CD8⁺ T cell function primarily by blocking inhibitory signaling pathways such as PD-1/PD-L1, thereby reinvigorating pre-existing effector CD8⁺ T cells [1]. In immunologically active hot tumors, this mechanism effectively releases the latent potential of antitumor immunity and leads to substantial clinical benefit.

However, in cold tumors, multiple hierarchical constraints—including priming failure, trafficking failure, and penetration failure—result in a markedly reduced baseline of tumor-specific CD8⁺ T cells. Under these conditions, even when inhibitory signals are relieved, the overall immune response may still remain below the immune activation threshold and fail to initiate sustained immune amplification. Thus, ICIs primarily function by releasing inhibitory “brakes,” rather than fundamentally lowering the immune activation threshold required for effective antitumor immune responses [21].

This limitation suggests that immune checkpoint blockade alone is insufficient to convert cold tumors into an immunologically active state. Therefore, effective immune remodeling strategies must accomplish two complementary objectives: first, to provide sufficient initial immune activation to drive immune responses across the immune activation threshold; and second, to establish intrinsic mechanisms capable of sustaining and amplifying antitumor immunity. In this context, immunoregulatory processes with amplification potential represent a critical entry point for promoting immune state transition.

## Ferroptosis as a threshold-sensitive trigger of immune initiation

### The functional basis of priming failure: limited antigen presentation efficiency

In cold tumors, priming failure does not primarily arise from a lack of tumor antigens, but rather reflects insufficient efficiency of antigen presentation [14, 15]. Under this constraint, the immune system may still recognize tumor-associated antigens; however, the resulting signal intensity remains inadequate to cross the immune activation threshold, thereby failing to effectively initiate downstream immune amplification.

Accordingly, overcoming priming failure is not simply a matter of increasing antigen abundance, but rather of enhancing the overall efficiency of antigen release, uptake, and presentation, such that immune input signals reach a level sufficient to trigger amplification. In this context, regulatory processes that simultaneously enhance antigen exposure and immune signaling input represent critical entry points for enabling the immune system to achieve threshold crossing.

### Ferroptosis enhances antigen exposure and immune signaling input

Ferroptosis is a regulated form of cell death driven by iron-dependent lipid peroxidation. Its relevance in immune regulation lies in its capacity to simultaneously provide antigenic input and danger-associated signals [9]. During ferroptosis, oxidative damage to membrane structures leads to the exposure or release of intracellular components into the extracellular space, including DAMPs [22].

For example, high-mobility group box 1 (HMGB1) can translocate from the nucleus and be released extracellularly, where it engages Toll-like receptor 4 (TLR4) or the receptor for advanced glycation end products (RAGE) on DCs, promoting nuclear factor κB (NF-κB) activation and favoring DC maturation and IL-12 production [23]. In addition, ATP released during the early phase of ferroptosis can activate the NLRP3 inflammasome via P2X7 receptors, leading to IL-1β secretion and further enhancing antigen presentation by DCs [24].

Collectively, these signals increase the likelihood that tumor antigens are effectively recognized and processed by the immune system, thereby strengthening input into the priming phase. From a functional perspective, ferroptosis should not be viewed merely as a form of cell death, but rather as a regulatory process that enhances the intensity of immune input signals, enabling otherwise insufficient antigenic stimuli to approach the activation threshold.

### Intrinsic constraints of ferroptosis: threshold dependence and limited amplification

Although ferroptosis can enhance immune-initiating signals, its effects are characterized by pronounced threshold dependence and temporal constraints. Moderate lipid peroxidation facilitates antigen exposure and DC activation, whereas excessive oxidative stress may generate high levels of reactive aldehydes, such as 4-hydroxynonenal (4-HNE), which can impair antigen-presenting cell function and potentially suppress immune responses [25]. In addition, key signaling molecules such as ATP are rapidly depleted during the later stages of ferroptosis, limiting the persistence of immune stimulation [26].

**Fig. 2:**
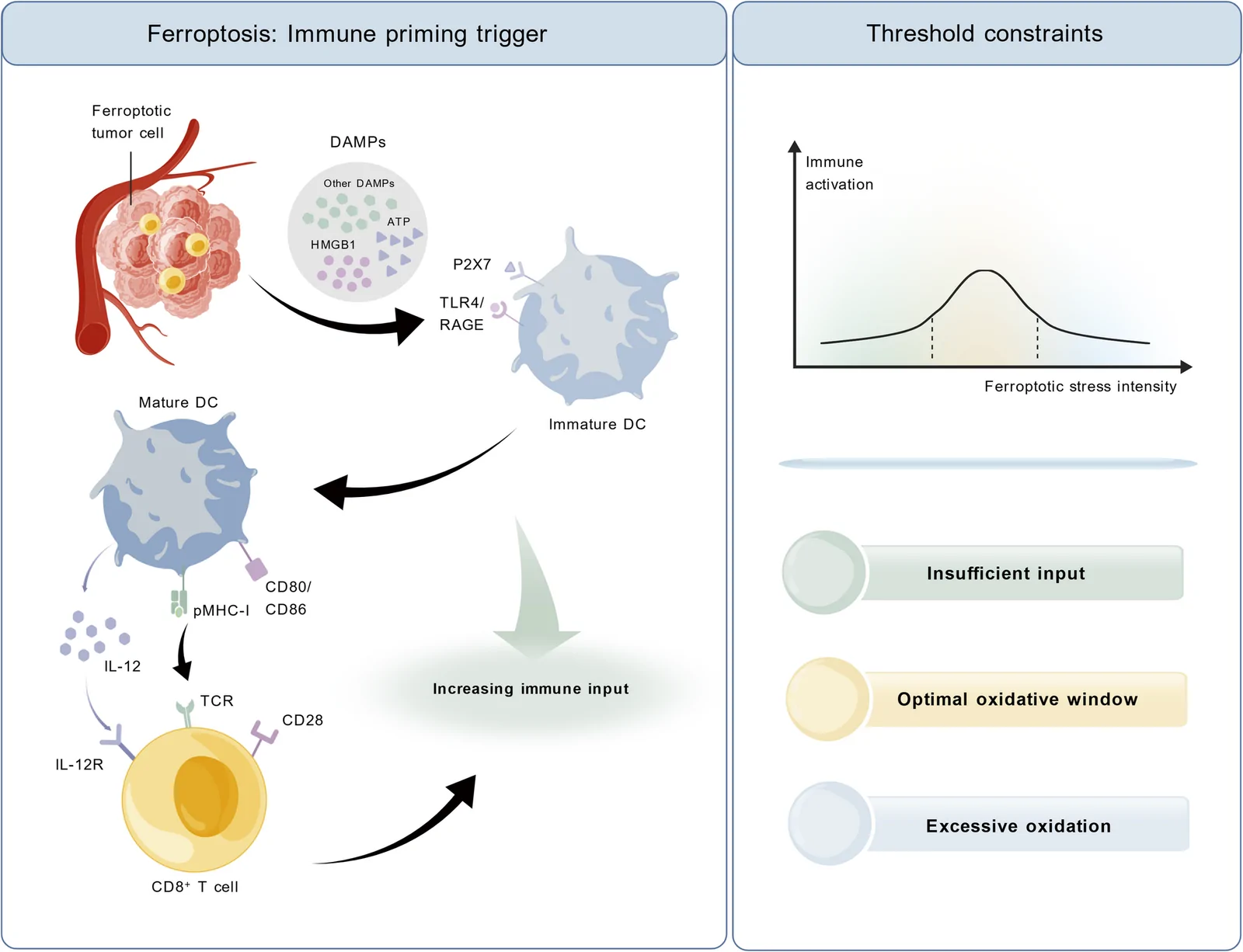
Ferroptosis as a threshold-sensitive trigger of immune priming. This figure illustrates ferroptosis as a key regulatory node in immune priming in cold tumors and highlights its threshold-dependent effects. On the left, ferroptotic tumor cells release damage-associated molecular patterns (DAMPs), including high-mobility group box 1 (HMGB1) and adenosine triphosphate (ATP). These signals are sensed by dendritic cells (DCs) via Toll-like receptor 4 (TLR4)/receptor for advanced glycation end products (RAGE) and P2X7 receptor (P2X7), respectively, promoting DC maturation and enhancing antigen presentation capacity, e.g., peptide–major histocompatibility complex class I (pMHC-I) expression and CD80/CD86 co-stimulatory signaling. In addition, DCs secrete interleukin-12 (IL-12), further supporting CD8⁺ T cell activation and effector differentiation, thereby facilitating the initiation of antitumor immune responses. Functionally, this process enhances antigen exposure and immune signaling input, enabling otherwise insufficient signals to approach or cross the immune activation threshold required for immune amplification. On the right, the immune effects of ferroptosis exhibit a pronounced intensity-dependent behavior. Low levels of oxidative stress provide insufficient immune input and fail to trigger effective immune responses. Moderate lipid peroxidation defines an optimal oxidative window that promotes DAMP release and DC activation, thereby facilitating immune priming. In contrast, excessive oxidative stress may generate toxic lipid peroxidation products and impair immune cell function, ultimately suppressing immune responses. Thus, ferroptosis acts as a threshold-sensitive process with biphasic regulatory effects, where its immunological outcome depends on the magnitude and temporal dynamics of oxidative stress

As a result, the immune input provided by ferroptosis is inherently transient and unstable, and is therefore insufficient on its own to drive sustained immune amplification. In this context, ferroptosis can be understood as a threshold-sensitive trigger of immune activation, capable of bringing immune signals close to—or transiently across—the immune activation threshold, but lacking the capacity to maintain and amplify this state. This intrinsic limitation implies that additional amplification mechanisms are required to convert initial immune inputs into stable antitumor responses (Fig. 2).

## STING signaling as an innate immune amplifier with context-dependent effects

### cGAS–STING converts DNA sensing into immune amplification

The cGAS–STING pathway is a central innate immune axis that mediates cytosolic DNA sensing and converts local DNA signals into systemic immune amplification [12]. In the tumor microenvironment, DNA fragments derived from tumor cells can accumulate in the cytosol due to cellular damage, stress, or aberrant division [27]. These DNA species are recognized by cGAS, which catalyzes the production of the second messenger cyclic GMP–AMP (cGAMP) [12].

cGAMP subsequently binds to STING on the endoplasmic reticulum membrane, inducing its activation and trafficking to the Golgi apparatus [12]. During this process, STING recruits and activates TANK-binding kinase 1 (TBK1) and IκB kinase (IKK), leading to the phosphorylation and nuclear translocation of interferon regulatory factor 3 (IRF3) and nuclear factor κB (NF-κB) [28].

This signaling cascade ultimately induces the expression of IFN-I and a range of inflammatory and chemotactic mediators. IFN-I promotes DC maturation and enhances cross-presentation, thereby facilitating CD8⁺ T cell activation and differentiation [29]. Meanwhile, NF-κB–dependent cytokines (e.g., TNF-α, IL-6, IL-12) support local inflammatory responses, and chemokines such as CXCL9 and CXCL10 establish gradients that drive effector CD8⁺ T cell recruitment [30].

Collectively, by coordinating antigen presentation and immune cell recruitment, the cGAS–STING pathway functions as a key amplifier that sustains and expands antitumor immune responses [31].

### Biphasic regulation of STING signaling: balancing amplification and suppression

Although the STING pathway exerts potent immunostimulatory effects, its function is highly dependent on the magnitude and duration of activation. Acute STING activation is typically associated with robust IFN-I production, promoting DC maturation and CD8⁺ T cell priming, thereby enhancing antitumor immunity [29].

In contrast, sustained or chronic activation may induce immunosuppressive feedback mechanisms, such as upregulation of indoleamine 2,3-dioxygenase (IDO) or increased PD-L1 expression, which can impair CD8⁺ T cell function and contribute to immune tolerance [32, 33]. This biphasic behavior suggests that STING does not function as a unidirectional amplifier, but rather as a tightly regulated signaling node.

In the context of cold tumors, this implies that STING activation alone, although capable of enhancing immune signaling, may be insufficient to sustain effective immune amplification without precise control of activation strength and duration, and may even shift toward immunosuppression under certain conditions.

### STING stabilizes threshold crossing from transient input to sustained amplification

Within an immune dynamical framework, the primary role of STING is not to initiate immune responses, but to stabilize and sustain immune states that are approaching or have crossed the activation threshold. Through IFN-I–dependent positive feedback, STING supports DC function and maintains chemokine expression, thereby promoting CD8⁺ T cell expansion and recruitment [29].

In contrast to ferroptosis, which provides transient immune input, STING primarily functions to convert these inputs into sustained immune amplification. However, in the absence of sufficient initial signals, STING activation alone may remain inadequate to drive the system across the immune activation threshold.

**Fig. 3:**
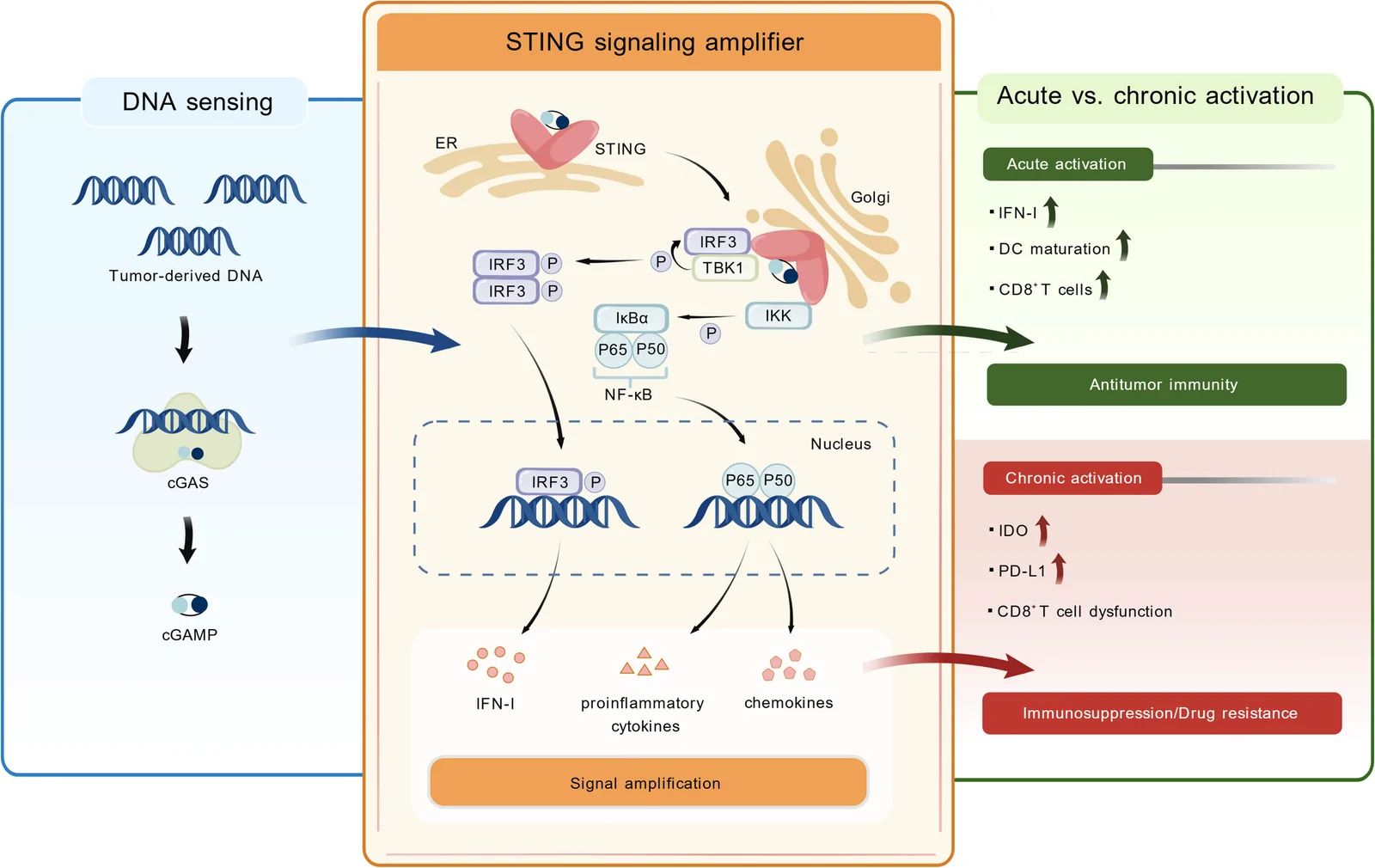
cGAS–STING signaling as an innate immune amplifier with context-dependent regulation. This figure illustrates the central role of the cyclic GMP–AMP synthase (cGAS)–stimulator of interferon genes (STING) pathway as an innate immune amplifier in the tumor microenvironment and its context-dependent regulatory features. On the left, tumor-derived DNA is sensed by cGAS, leading to the production of cyclic GMP–AMP (cGAMP), which subsequently activates STING on the endoplasmic reticulum (ER) and induces its trafficking to the Golgi apparatus. During this process, STING recruits and activates TANK-binding kinase 1 (TBK1) and IκB kinase (IKK), thereby driving interferon regulatory factor 3 (IRF3) and nuclear factor-κB (NF-κB) signaling. Activated IRF3 and NF-κB translocate into the nucleus and induce the expression of type I interferons (IFN-I), inflammatory cytokines, and chemokines such as CXCL9 and CXCL10. Functionally, this signaling cascade amplifies innate immune responses by promoting dendritic cell (DC) maturation and facilitating CD8⁺ T cell activation and recruitment. On the right, STING signaling exhibits pronounced context-dependent and biphasic regulatory behavior. Acute activation enhances IFN-I production, promotes DC maturation, and supports CD8⁺ T cell responses, thereby facilitating antitumor immunity. In contrast, sustained or chronic activation may induce immunosuppressive programs, including upregulation of indoleamine 2,3-dioxygenase (IDO), increased programmed death ligand 1 (PD-L1) expression, and impairment of CD8⁺ T cell function, ultimately contributing to immune suppression or therapeutic resistance. Thus, STING signaling functions not as a unidirectional immunostimulatory pathway, but as a tightly regulated signaling node whose outcome depends on the magnitude and duration of activation, balancing immune amplification and immune suppression

This suggests a potential functional complementarity between ferroptosis-driven immune input and STING-mediated amplification. Whether these processes can couple to enable a transition from a near-threshold to a threshold-crossing immune state represents a key question for further investigation (Fig. 3).

## The ferroptosis–cGAS–STING amplification circuit

Within the immune threshold framework described above, we propose the concept of the Ferroptosis–cGAS–STING amplification circuit, which captures a context-dependent and transient coupling between ferroptotic stress and innate immune sensing. Rather than representing a constitutively active signaling pathway, this circuit is better understood as a functional amplification module that depends on specific temporal windows and metabolic conditions, enabling subthreshold immune responses to cross the immune activation threshold.

### Ferroptosis as an upstream trigger of cGAS–STING activation

Accumulating evidence suggests that, under specific conditions, ferroptosis-associated oxidative stress can act as an upstream source of signals for cGAS–STING activation. In addition to enhancing immune input through DAMP release and lipid peroxidation, ferroptosis-associated mitochondrial damage and DNA leakage provide a potential molecular basis for cytosolic DNA sensing [34].

During ferroptosis, iron-dependent Fenton reactions drive the accumulation of reactive oxygen species (ROS), leading to oxidative damage of mitochondrial membranes enriched in polyunsaturated fatty acids (PUFAs) [35]. ROS can also induce mitochondrial DNA (mtDNA) oxidation, such as the formation of 8-oxo-deoxyguanosine (8-oxo-dG). When the extent of damage exceeds mitochondrial base excision repair capacity, oxidized mtDNA fragments may be released into the cytosol, where they can be recognized by cGAS, thereby activating STING signaling and inducing IFN-I production [36].

This process is further modulated by cellular iron metabolism. Upregulation of nuclear receptor coactivator 4 (NCOA4) promotes ferritinophagy, leading to iron overload and mitochondrial damage, thereby facilitating mtDNA release [37]. Similarly, increased expression of transferrin receptor 1 (TFR1) enhances iron uptake, contributing to oxidative stress and DNA release [38]. Thus, iron metabolism not only determines ferroptosis susceptibility but may also influence the magnitude of DNA sensing.

Importantly, the impact of ferroptosis on STING signaling is both dose- and time-dependent. Moderate lipid peroxidation may facilitate DNA release and signal initiation, whereas excessive lipid peroxidation products, such as 4-HNE, can modify critical cysteine residues on STING (e.g., Cys88), impairing its palmitoylation and trafficking, and thereby attenuating downstream signaling [39]. In contrast, glutathione peroxidase 4 (GPX4)-mediated clearance of lipid peroxides may help preserve STING functional integrity [39, 40].

In addition, the cellular source of STING activation may vary depending on the context. DNA signals may activate cGAS–STING signaling within tumor cells, or alternatively be released and subsequently taken up by antigen-presenting cells such as DCs, thereby activating host immune pathways [41, 42]. This suggests that the coupling between ferroptosis and STING signaling is likely cell type-dependent.

Overall, current evidence supports ferroptosis as a conditional upstream trigger of cGAS–STING activation, the magnitude of which is jointly determined by the level of oxidative stress, temporal dynamics, and the cellular context. Importantly, this regulation is not unidirectional. Increasing evidence indicates that STING signaling can, in turn, reshape cellular susceptibility to ferroptosis, thereby establishing a potential bidirectional regulatory relationship.

### STING signaling reinforces ferroptotic vulnerability

Complementary to the upstream input provided by ferroptosis, accumulating evidence suggests that, under specific conditions, cGAS–STING signaling may enhance cellular susceptibility to ferroptosis through multiple mechanisms, thereby establishing a potential positive-feedback interaction.

At the level of lipid metabolism, STING has been reported to interact with acyl-CoA synthetase long-chain family member 4 (ACSL4), a key enzyme that determines ferroptosis susceptibility by promoting the incorporation of PUFAs into phospholipids, generating lipid species prone to peroxidation [43, 44]. This STING–ACSL4 axis may increase the availability of substrates for lipid peroxidation, thereby enhancing ferroptotic sensitivity [43].

At the level of antioxidant regulation, STING can influence cellular redox balance through IFN-I signaling. In head and neck squamous cell carcinoma (HNSCC) models, STING activation has been shown to downregulate xCT (SLC7A11) and GPX4 via IFN-I signaling, leading to reduced cystine uptake and glutathione synthesis, thereby impairing the cellular capacity to detoxify lipid peroxides and increasing susceptibility to ferroptosis [45]. Notably, ferroptosis itself may further amplify STING–IFN-I signaling, suggesting a potential feedback interaction [45].

In addition, STING may modulate oxidative stress through mitochondrial mechanisms. Under ferroptotic conditions, STING has been observed to localize to mitochondria and interact with mitofusin 1/2 (MFN1/2), promoting mitochondrial fusion and ROS production [46]. Loss of STING or MFN1/2 has been associated with reduced lipid peroxidation and diminished ferroptosis sensitivity [46].

Collectively, current evidence supports a bidirectional regulatory relationship between ferroptosis and cGAS–STING signaling, which provides the molecular basis for the subsequent immune amplification process. However, this bidirectional interaction is currently supported primarily by mechanistic studies conducted in distinct experimental models. Whether it is consistently conserved across different tumor types and capable of establishing a stable feedback amplification loop remains to be further validated.

### Immune setpoint reset through a transient ferroptosis–cGAS–STING amplification circuit

Building upon the bidirectional regulatory relationship between ferroptosis and cGAS–STING signaling described in Sect. 4.1 and 4.2, we propose that, under appropriate conditions, this system functions as a transient amplification circuit that links ferroptosis-associated danger-signal input to innate immune amplification. Constrained by temporal and metabolic conditions, this circuit may regulate the threshold-crossing behavior of immune responses in cold tumors.

Within the threshold-constrained immune state of cold tumors, the tumor–immune system remains in a low-intensity steady state that is unable to sustain itself, causing immune responses to persist below the immune activation threshold [21]. This state reflects a functional mismatch between antigenic input and immune amplification. Consequently, neither ferroptosis induction nor cGAS–STING activation alone is sufficient to generate durable antitumor immunity. Ferroptosis primarily provides transient antigenic and danger-signal inputs but lacks stable downstream amplification, whereas cGAS–STING activation can initiate IFN-I-dependent immune programs but may fail to sustain them in the absence of sufficient and persistent upstream DNA-derived signals.

Within this context, transient coupling between ferroptosis and cGAS–STING signaling may generate conditional immune amplification during a defined temporal window. Ferroptosis-associated stress promotes the release of mtDNA and other DNA-derived signals into the cytosol, thereby activating the cGAS–STING–IFN-I axis. In turn, STING signaling may enhance cellular susceptibility to ferroptosis through regulation of the xCT–GPX4 axis, ACSL4-dependent lipid remodeling, and ROS production, thereby facilitating subsequent DNA-derived signal input. Together, these processes constitute the functional manifestation of the bidirectional regulatory relationship described above [29, 36, 43, 45, 46].

Nevertheless, this amplification process is more likely to function as a temporally restricted and transient regulatory event rather than a sustained positive-feedback state [47]. On the one hand, as upstream DNA-derived signals diminish and oxidative stress subsides, immune amplification gradually declines, making prolonged high-level signaling difficult to maintain [48]. On the other hand, persistent or excessive STING activation may induce chronic inflammation and immune dysregulation, whereas excessive ferroptosis may impair antitumor immune cells, including DCs and effector CD8⁺ T cells, thereby limiting subsequent immune amplification [49–51].

**Fig. 4:**
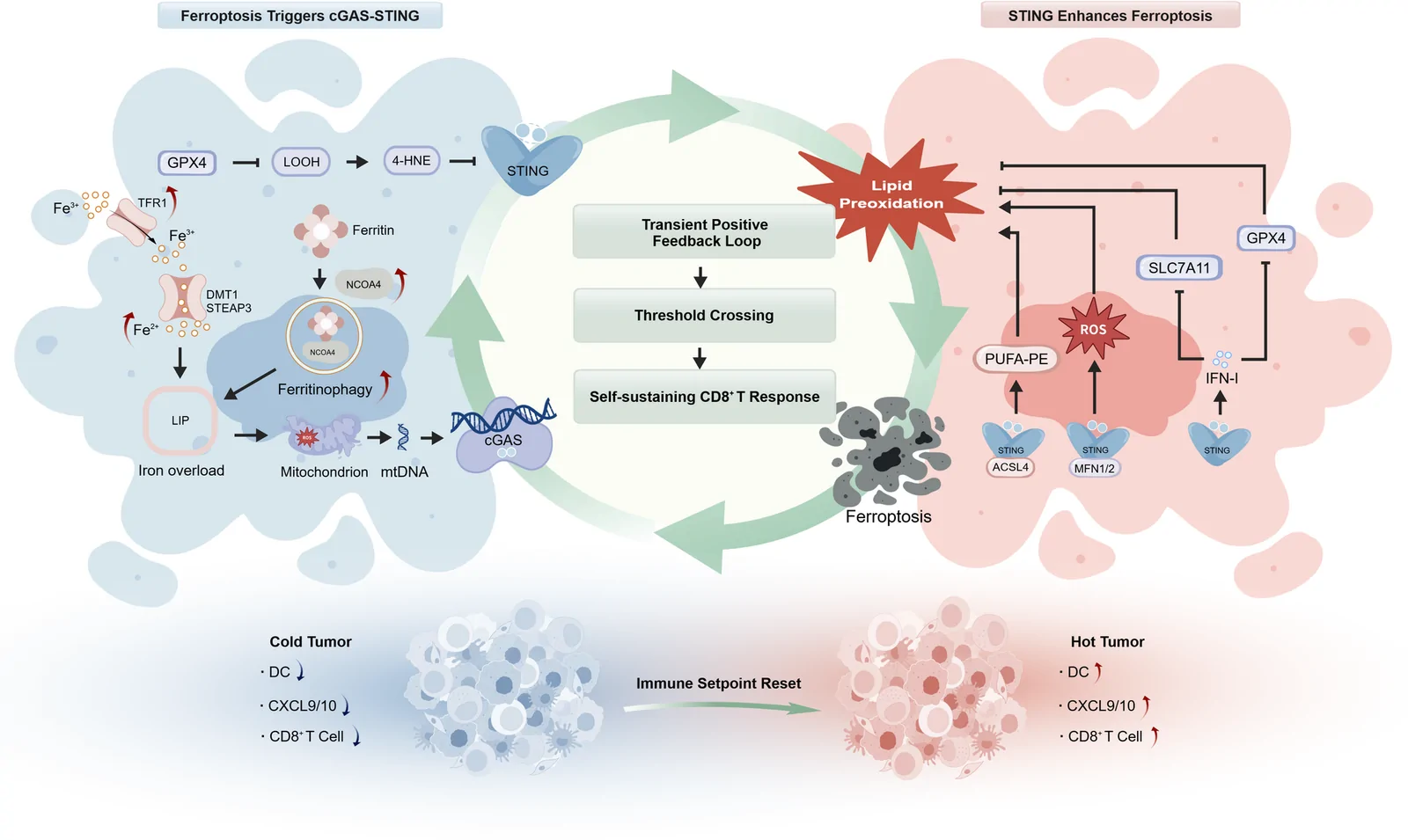
The ferroptosis–cGAS–STING amplification circuit drives threshold crossing and immune setpoint reset. This figure illustrates a context-dependent Ferroptosis–cyclic GMP–AMP synthase (cGAS)–stimulator of interferon genes (STING) amplification circuit that enables threshold crossing and immune setpoint resetting in cold tumors. On the left, ferroptosis initiates mitochondrial damage and mitochondrial DNA (mtDNA) release, which is sensed by cGAS to activate STING signaling. In parallel, upregulation of transferrin receptor 1 (TFR1) enhances iron uptake, whereas increased nuclear receptor coactivator 4 (NCOA4)-mediated ferritinophagy promotes iron release; together, these processes elevate intracellular labile iron and oxidative stress, further facilitating mitochondrial damage and DNA release. Within this context, excessive lipid peroxidation products such as 4-hydroxynonenal (4-HNE) may suppress STING activity, whereas glutathione peroxidase 4 (GPX4) limits lipid peroxidation through detoxification of lipid hydroperoxides (LOOH), thereby preserving STING signaling. On the right, STING signaling reinforces ferroptotic vulnerability through multiple parallel mechanisms, including increased availability of peroxidizable lipids via acyl-CoA synthetase long-chain family member 4 (ACSL4), suppression of the solute carrier family 7 member 11 (SLC7A11/xCT)–GPX4 antioxidant axis through type I interferon (IFN-I) signaling, and enhanced mitochondrial reactive oxygen species (ROS) production mediated by mitofusin 1/2 (MFN1/2). These processes collectively amplify oxidative stress and ferroptosis-associated signaling input. At the center, these bidirectional interactions form a transient positive feedback loop. Within an appropriate temporal and metabolic window, ferroptosis-derived DNA and oxidative inputs are converted by cGAS–STING signaling into an amplifiable immune state, while STING-mediated reinforcement of ferroptotic sensitivity sustains this process, enabling subthreshold immune activity to achieve threshold crossing. This transition supports the establishment of a self-amplifying antitumor program, conceptualized as immune setpoint resetting. At the bottom, the circuit-driven transition from cold to immune-active tumors is illustrated. Cold tumors are characterized by low levels of dendritic cells (DCs), CXCL9/CXCL10, and CD8⁺ T cells. Activation of the amplification circuit promotes the coordinated increase of these immune components, driving the transition toward an immune-active tumor state

Therefore, the biological significance of this circuit may lie not in maintaining persistently elevated immune activation, but rather in transiently reshaping the functional state of the immune system. By enabling the transition from a subthreshold immune steady state to a transiently suprathreshold amplification state through crossing the immune activation threshold, this circuit may establish conditions that favor the emergence of a new immune steady state. In this Review, we refer to this continuous dynamic process—initiated by crossing the immune activation threshold and culminating in functional remodeling of the tumor–immune system—as immune setpoint resetting (Fig. 4).

## Multilevel alleviation of immune failure in cold tumors via the ferroptosis–cGAS–STING circuit

Immune resistance in cold tumors is typically maintained by hierarchical layers of immune failure, including priming failure, trafficking failure, and penetration failure. A single intervention is often insufficient to establish stable immune amplification across multiple levels simultaneously. By simultaneously providing antigenic and DNA-derived inputs and facilitating the integration of these signals into an amplifiable immune program through IFN-I signaling, the Ferroptosis–cGAS–STING amplification circuit may cooperatively alleviate immune constraints across multiple levels.

### Restoration of priming capacity

Priming failure primarily reflects insufficient cross-presentation capacity and unstable DC maturation, thereby limiting the effective expansion of CD8⁺ T cells. The ferroptosis–cGAS–STING axis may simultaneously provide antigenic and DNA-derived inputs, activate IFN-I signaling, and promote DC maturation, thereby strengthening the priming process.

In HNSCC models, ferroptosis induction activates the cGAS–STING–IFN-I axis, enhancing DC maturation and CD8⁺ T cell infiltration. IFN-I further suppresses the xCT (SLC7A11)–GPX4 antioxidant axis, reducing cellular buffering capacity against lipid peroxidation and prolonging ferroptosis-associated signaling input. In vivo, combined ferroptosis induction and STING activation increase intratumoral DC and CD8⁺ T cell accumulation and exhibit superior antitumor efficacy compared with either monotherapy [45].

Similar mechanisms have also been demonstrated in nanodelivery systems. For example, the HBMn-FA platform induces ferroptosis-associated mtDNA release, whereas Mn²⁺ enhances cGAS-mediated DNA sensing, thereby synergistically activating STING and upregulating IFN-I. This promotes DC maturation, antigen-specific CD8⁺ T cell expansion, and improves responses to ICIs [52].

Collectively, current preclinical evidence supports a role for the ferroptosis–cGAS–STING axis in reinforcing immune priming. Ferroptosis provides antigenic and DNA-derived inputs, whereas STING converts these signals into an IFN-I-driven priming program, thereby facilitating the transition from inefficient immune initiation toward an amplifiable immune state.

### Reconstitution of T cell trafficking

Trafficking failure primarily results from insufficient chemokine gradients, thereby limiting the recruitment of primed CD8⁺ T cells into the tumor microenvironment. In particular, the CXCL10–CXCR3 axis plays a central role in directing effector CD8⁺ T cell trafficking [53]. Therefore, effective restoration of CD8⁺ T cell recruitment requires re-establishment of this chemokine network.

The ferroptosis–cGAS–STING axis provides mechanistic support for this process. In a Gpx4-deficient hepatocellular carcinoma model, ferroptosis induces oxidative DNA damage and activates cGAS–STING signaling, leading to marked upregulation of CXCL10. This effect is STING-dependent, as pharmacological inhibition of STING markedly attenuates ferroptosis-induced CXCL10 production [54]. Elevated CXCL10 establishes a CXCR3-dependent chemotactic gradient that promotes CD8⁺ T cell infiltration, whereas deletion of CXCR3 abolishes this recruitment effect [54].

Collectively, available evidence suggests that the ferroptosis–cGAS–STING axis can promote effector CD8⁺ T cell recruitment by restoring the CXCL10–CXCR3 chemokine axis. However, this conclusion is currently supported primarily by a limited number of tumor models, and its applicability across diverse cold tumor settings remains to be established.

### Relief of spatial exclusion and improvement of tumor penetration

Penetration failure primarily arises from structural constraints within the tumor stroma, including CAF-driven ECM deposition, TGF-β-mediated stromal remodeling, and aberrant vascular architecture [55, 56]. Under specific conditions, the ferroptosis–cGAS–STING axis may modulate stromal and vascular states, thereby creating a more permissive microenvironment for effector CD8⁺ T cell infiltration into the tumor parenchyma.

In vivo studies have shown that near-infrared II (NIR-II)-triggered ferroptosis combined with STING agonist-based nanoplatforms reduces abnormal vascular density, improves tissue architecture, and enhances CD8⁺ T cell infiltration [57]. These findings suggest that ferroptosis-derived DNA and associated danger signals may cooperate with STING-mediated IFN-I amplification to contribute to remodeling of the tumor microenvironment.

However, current evidence is derived primarily from functional readouts, including CD8⁺ T cell infiltration and antitumor efficacy, whereas direct evidence regarding CAF activation states, ECM remodeling, and spatial tissue architecture remains limited. Accordingly, the available data are insufficient to conclude that the ferroptosis–cGAS–STING axis broadly overcomes stromal barriers.

Mechanistically, emerging studies suggest that ferroptosis may modulate TGF-β–CAF signaling through lipid peroxidation and oxidative stress, whereas STING activation may improve endothelial function and promote vascular normalization via IFN-I signaling, thereby providing a potential mechanistic basis for enhanced tissue perfusion and CD8⁺ T cell entry into the tumor core [58–61].

Collectively, current evidence suggests that the ferroptosis–cGAS–STING axis may contribute to remodeling of the tumor microenvironment and thereby facilitate relief of spatial exclusion. However, its capacity to improve tumor penetration and broadly overcome stromal barriers remains largely inferential and has not yet been directly demonstrated across diverse tumor settings (Table 1).

**Table 1:** Representative studies supporting ferroptosis–cGAS–STING coupling across distinct layers of immune repair

Study	Tumor model	Intervention	Key mechanistic finding	Immune repair outcome (functional layer)	Level of immune repair
Li et al., 2025	HNSCC mouse model	Ferroptosis induction combined with STING agonist	Ferroptosis activates cGAS–STING–IFN-I signaling, which suppresses the xCT–GPX4 axis	Enhanced DC maturation and CD8⁺ T cell priming	Priming
Liang et al., 2023	HCC-bearing mouse model	Mn²⁺-assisted ferroptosis and cGAS–STING activation	Ferroptosis-derived mtDNA activates cGAS–STING signaling	Enhanced DC maturation and CD8⁺ T cell priming	Priming
Conche et al., 2022	HCC mouse model	GPX4 deficiency-induced ferroptosis	Ferroptosis-induced DNA damage drives cGAS–STING-dependent CXCL10 expression	Increased CXCR3-dependent CD8⁺ T cell recruitment	Trafficking
Zhao et al., 2025	Orthotopic 4T1 and B16-F10 mouse models	Fe-THBQ/SR with 1064 nm irradiation	ROS-driven ferroptosis and dsDNA leakage cooperate with SR-717 to amplify STING activation, normalize vasculature, and relieve hypoxia	Improved CD8⁺ T cell infiltration into tumors	Penetration

Note: Immune repair levels are defined as priming (dendritic cell activation and T cell priming), trafficking (T cell recruitment), and penetration (tumor infiltration)

Abbreviations: cGAS, cyclic GMP–AMP synthase; STING, stimulator of interferon genes; HNSCC, head and neck squamous cell carcinoma; HCC, hepatocellular carcinoma; DC, dendritic cell; IFN-I, type I interferon; GPX4, glutathione peroxidase 4; xCT/SLC7A11, solute carrier family 7 member 11; mtDNA, mitochondrial DNA; dsDNA, double-stranded DNA; ROS, reactive oxygen species; CXCL9/10, C-X-C motif chemokine ligand 9/10; CXCR3, C-X-C motif chemokine receptor 3

## The ferroptosis–cGAS–STING amplification axis: regulated dynamics and translational strategies

Within the immunodynamic framework described above, the ferroptosis–cGAS–STING axis can be conceptualized as a context-dependent immune amplification module, whose functional potential in cold tumors depends on the precise regulation of signal intensity, temporal dynamics, and spatial distribution. Through the coupling of ferroptosis-associated antigenic and DNA-derived inputs with innate immune signaling, this system may, under permissive conditions, facilitate the transition of immune responses across the activation threshold, shifting from a low-intensity state toward a more amplifiable immune program.

However, this amplification process exhibits a clear “double-edged” nature. In the absence of appropriate regulation, it may lead to oxidative damage, chronic inflammation, or even the induction of immunosuppressive programs. Therefore, the translational application of this axis relies on achieving a balance between immune activation and system stability, and on the establishment of a controllable amplification window defined by signal intensity, temporal dynamics, and spatial confinement, in conjunction with patient stratification and treatment sequencing strategies.

### Signal intensity: a narrow functional window

At the level of signal intensity, ferroptosis-associated immune modulation displays a pronounced dose-dependent effect. Moderate lipid peroxidation may enhance antigen exposure and provide DNA-derived inputs that support immune activation, whereas excessive oxidative stress can impair antigen-presenting cells, reduce T cell viability, and increase systemic toxicity risk [62]. Similarly, STING agonists exhibit biphasic effects: excessive activation may induce inflammatory toxicity, while insufficient activation may fail to achieve effective IFN-I amplification [63]. In a phase I clinical study, the STING agonist MIW815 (ADU-S100) was also limited by safety concerns and a narrow therapeutic window [64].

Accordingly, translational strategies may be designed around the principle of minimal sufficient gain, aiming to achieve threshold crossing while avoiding oxidative overload and systemic inflammatory risk, particularly in patient populations with substantial heterogeneity in ferroptosis susceptibility and STING signaling status.

### Temporal dynamics: transient activation versus chronic stimulation

From a temporal perspective, ferroptosis-associated signals often exhibit pulsatile dynamics. Early-phase release of ROS, DAMPs, and DNA can facilitate DNA sensing and IFN-I amplification, whereas sustained oxidative stress may impair antigen-presenting cell function and disrupt signal transduction [65]. STING signaling also displays biphasic dynamics: acute activation promotes dendritic cell maturation and CD8⁺ T cell expansion, whereas chronic activation may induce immunoregulatory programs, leading to an “inflamed-but-suppressed” state [66].

Accordingly, from a translational standpoint, this amplification system may benefit more from short-term activation combined with intermittent stimulation strategies, such as pulsed induction of ferroptosis or staged activation of STING signaling. Such temporally structured approaches may help maintain immune activation while minimizing chronic inflammation-associated negative feedback.

### Spatial confinement: local amplification and control of systemic risk

At the spatial level, IFN-I signaling mediated by the cGAS–STING pathway has the potential to diffuse beyond the local microenvironment [41]. Systemic activation may trigger widespread inflammation and induce immunosuppressive responses, thereby attenuating local antitumor effects. Therefore, in clinical applications, the amplification process should be preferentially confined to the tumor microenvironment and its draining lymph nodes.

Emerging evidence suggests that spatially restricted strategies—including intratumoral delivery, tumor-targeted nanocarriers, and region-specific immunomodulatory platforms—may enable localized activation of the ferroptosis–STING axis. These approaches may enhance local immune amplification while reducing systemic toxicity, and are likely to represent a critical prerequisite for the successful clinical translation of this amplification system [67–69].

### Patient stratification: pathway competence as a determinant of response

The effectiveness of ferroptosis–cGAS–STING–mediated amplification depends on the functional integrity of the underlying pathways, which can vary substantially across patients. For example, certain tumors may exhibit ferroptosis resistance (e.g., high SLC7A11/GPX4 expression), STING pathway silencing (e.g., epigenetic suppression), impaired IFN-I responsiveness, or deficient chemokine expression, thereby limiting effective immune amplification [41, 70–73].

Therefore, patient stratification based on pathway status is of considerable importance. Potential biomarkers may include ferroptosis-related regulators (such as SLC7A11 and GPX4), expression or activation status of the cGAS–STING axis, IFN-response gene signatures, and chemokine programs such as CXCL9/CXCL10. These indicators may help identify patient subgroups with a higher likelihood of responding to amplification-based strategies, thereby improving therapeutic efficacy and reducing unnecessary interventions.

### Sequential coordination with immune checkpoint inhibitors

In combination strategies involving ICIs, the ferroptosis–cGAS–STING axis and ICIs may exhibit mechanistic complementarity. The former primarily contributes to immune initiation by enhancing antigen presentation and chemokine production, thereby promoting T cell recruitment, whereas ICIs restore T cell effector function by blocking inhibitory pathways such as PD-1/PD-L1 [74].

Based on this division of roles, a potentially rational sequential strategy is to first engage the ferroptosis–STING axis to enhance immune priming and T cell infiltration, followed by the administration of ICIs to sustain and amplify antitumor immune responses. This approach may better align with the dynamic nature of immune activation, although the optimal timing, dosing, and patient selection remain to be determined.

## Limitations and future directions

Despite providing a unified threshold-based framework for ferroptosis–cGAS–STING coupling in cold tumors, several limitations specific to the current evidence base should be acknowledged.

First, the proposed system-level model is primarily supported by preclinical studies conducted in heterogeneous genetic and metabolic contexts. As a result, whether a stable and reproducible ferroptosis–cGAS–STING amplification circuit exists across diverse tumor types remains uncertain. In particular, heterogeneity in ferroptosis sensitivity (e.g., SLC7A11–GPX4 axis activity) and variability in STING pathway integrity may critically influence whether this system functions as an effective threshold-crossing amplifier or remains a context-dependent, transient interaction [35, 41, 51].

Second, evidence supporting downstream effects beyond immune activation—particularly in tumor penetration and stromal remodeling—is largely indirect. Observations related to CAFs, ECM remodeling, and vascular normalization are primarily inferred from immune infiltration phenotypes rather than directly quantified changes in spatial tissue architecture. Therefore, current data remain insufficient to conclude that the ferroptosis–cGAS–STING axis broadly and consistently resolves physical stromal barriers across tumor types [19, 20].

Third, the immune setpoint resetting concept proposed in this Review represents a systems-level hypothesis describing emergent immune state transitions rather than a directly validated biological state. Key properties of this transition, including the temporal boundaries of the amplification window and the stability of the post-transition immune configuration, remain to be experimentally defined.

Future studies integrating spatial transcriptomics, multiplex imaging, lineage-resolved functional perturbation, and time-resolved immune profiling will be required to determine whether ferroptosis–cGAS–STING coupling can reliably induce threshold-crossing behavior and durable immune state reprogramming across heterogeneous tumor microenvironments.

## Conclusion

Cold tumors have traditionally been regarded as tumors characterized by limited immune cell infiltration and weak inflammatory signaling. In this Review, however, we propose that many cold tumors are not completely immunologically inert but are more appropriately understood as a threshold-constrained immune state. Within this state, antitumor immune responses are not absent but instead persist at a subthreshold level below the immune activation threshold required for effective immune amplification. Hierarchical immune barriers—including priming failure, trafficking failure, and penetration failure—collectively constrain sustained amplification of the cancer–immunity cycle, thereby maintaining the tumor–immune system in a low-responsive steady state.

Based on this perspective, we propose the Ferroptosis–cGAS–STING amplification circuit as a conceptual framework linking ferroptosis-associated antigenic and DNA-derived signals to innate immune activation. We propose that this circuit is more appropriately understood as a temporally restricted transient amplification module rather than a sustained positive-feedback system. Under appropriate conditions, this transient immune amplification may enable immune responses that would otherwise remain subthreshold to cross the immune activation threshold, thereby creating conditions favorable for subsequent immune amplification.

Furthermore, we propose that the biological significance of this circuit lies not in maintaining persistently elevated immune activation but in driving the functional transition of the tumor–immune system from a low-responsive state toward a more immunocompetent state. We define this systemic functional remodeling, initiated by crossing the immune activation threshold, as immune setpoint resetting. This concept emphasizes the dynamic reprogramming of the overall functional state of the tumor–immune system rather than the sustained activation of an individual signaling pathway. Although this conceptual framework requires further validation across diverse tumor contexts, it not only provides a unified systems-level perspective for understanding immune remodeling in cold tumors but also offers a theoretical foundation for the rational design of threshold-based immunotherapeutic strategies.

## Data Availability

Not applicable.
